# Impact of arrhythmogenic calmodulin variants on small conductance Ca^2+^‐activated K^+^ (SK3) channels

**DOI:** 10.14814/phy2.14210

**Published:** 2019-10-06

**Authors:** Arnela Saljic, Kalai Mangai Muthukumarasamy, Jonas Marstrand la Cour, Kim Boddum, Morten Grunnet, Martin Werner Berchtold, Thomas Jespersen

**Affiliations:** ^1^ Laboratory of Cardiac Physiology, Faculty of Health and Medical Sciences, Department of Biomedical Sciences University of Copenhagen Copenhagen Denmark; ^2^ Cell Biology and Physiology, Department of Biology University of Copenhagen Copenhagen Denmark; ^3^ H. Lundbech A/S Valby Denmark

**Keywords:** Calmodulin, cardiac arrhythmias, channelopathies, CPVT, LQTS, small conductance Ca^+^‐activated K^+^ channels

## Abstract

Calmodulin (CaM) is a ubiquitous Ca^2+^‐sensing protein regulating many important cellular processes. Several CaM‐associated variants have been identified in a small group of patients with cardiac arrhythmias. The mechanism remains largely unknown, even though a number of ion channels, including the ryanodine receptors and the L‐type calcium channels have been shown to be functionally affected by the presence of mutant CaM. CaM is constitutively bound to the SK channel, which underlies the calcium‐gated *I*_SK_ contributing to cardiac repolarization. The CaM binding to SK channels is essential for gating, correct assembly, and membrane expression. To elucidate the effect of nine different arrhythmogenic CaM variants on SK3 channel function, HEK293 cells stably expressing SK3 were transiently co‐transfected with CaM^WT or variant^ and whole‐cell patch‐clamp recordings were performed with a calculated free Ca^2+^ concentration of 400 nmol/L. MDCK cells were transiently transfected with SK3 and/or CaM^WT or variant^ to address SK3 and CaM localization by immunocytochemistry. The LQTS‐associated variants CaM^D96V^, CaM^D130G^, and CaM^F142L^ reduced *I*_SK,Ca_ compared with CaM^WT^ (*P* < 0.01, *P* < 0.001, and *P* < 0.05, respectively*).* The CPVT associated variant CaM^N54I^ also reduced the *I*_SK,Ca_ (*P* < 0.05), which was linked to an accumulation of SK3/CaM^N54I^ channel complexes in intracellular compartments (*P* < 0.05). The CPVT associated variants, CaM^A103V^ and CaM^D132E^ only revealed a tendency toward reduced current, while the variants CaM^F90L^ and CaM^N98S^, causing LQTS syndrome, did not have any impact on *I*_SK,Ca_. In conclusion, we found that the arrhythmogenic CaM variants CaM^N54I^, CaM^D96V^, CaM^D130G^, and CaM^F142L^ significantly down‐regulate the SK3 channel current, but with distinct mechanism.

## Introduction

The family of small conductance Ca^2+^‐activated K^+^ (SK) channels consists of three subtypes, namely SK1 (*KCNN1*), SK2 (*KCNN2*), and SK3 (*KCNN3*) (Wei 2005). SK channels are primarily recognized for their wide distribution in the central nervous system, where they contribute to the control of neuronal excitability by mediating the afterhyperpolarization of medium duration (Kohler 1996; Vergara 1998; Stocker and Pedarzani 2000; Stocker 2004). Over the last years an increasing amount of evidence has been accumulated, showing the functional importance of SK channels in the heart, where they in particular contribute to atrial repolarization (Tuteja 2005; Zhang 2014). Under normal physiological conditions the functional importance of SK channels in humans is greater in atrial than ventricular tissue. This atrial selectivity makes SK channels an interesting target in the treatment of atrial arrhythmias, as the ventricular side effects have been speculated to be avoided (Xu 2003; Diness 2010, 2017; Skibsbye 2011; Skibsbye 2014). However, several recent studies have shown that SK channel current also becomes functional in the ventricles during enhanced sympathetic activation through *β*‐adrenergic stimulation (Chen 2018; Hamilton 2019).

Functional SK channels are composed of four α‐subunits with six transmembrane segments with both N‐ and C‐termini located intracellularly. Even though SK channels exhibit a topology similar to the voltage‐gated K^+^ channels, they are nonetheless voltage‐independent and are solely gated by the rise of intracellular Ca^2+^ (Vergara 1998; Xia 1998; Stocker 2004). All three subtypes of SK channels have similar Ca^2+^ dose‐response relationships with half maximal activation (*K*_0.5_) obtained at approximately 300 nmol/L Ca^2+^ and a Hill coefficient of 4‐5 (Xia 1998). The primary structure of SK channels does not reveal an obvious Ca^2+^‐binding domain. Instead, the C‐termini of all SK channel subtypes constitutively interact with the intracellular Ca^2+^ sensor calmodulin (CaM) through a CaM‐binding domain (CaMBD) (Xia 1998). The CaMBD of SK channels stretches over approximately 90 amino acids and is found at the C‐terminus just adjacent to the S6 transmembrane segment (Schumacher 2001). CaM is a highly conserved 148‐amino acid protein composed of an N‐ and C‐terminus each containing a pair of EF‐hands connected by a flexible linker. Each of these four EF‐hands bind one Ca^2+^ (Marshall 2015). The SK/CaM interaction is both Ca^2+^‐dependent and – independent (Schumacher 2001; Villalobo 2018). The C‐lobe of CaM and the flexible linker region have been proposed to be responsible for the constitutive interaction between CaM and the CaMBD of SK channels in the absence of Ca^2+^ (Keen 1999; Schumacher 2001). Binding of Ca^2+^ to the N‐lobe of CaM leads to a conformational change as two CaMBD/CaM dimers form and create a rotary force that is transmitted to the S6 pore helices, resulting in the opening of the channel (Schumacher 2001). In addition to its essential role in channel gating, CaM also seems to play an important role in SK channel assembly and trafficking to the plasma membrane. SK2 channels lacking the Ca^2+^‐independent constitutive CaMBD/CaM interaction are not trafficked to the surface membrane, but are retained in the Golgi complex (Lee 2003; Roncarati et al. 2005). Although the CaMBD of SK channels is necessary for channel trafficking it is not sufficient, as also downstream sequences of the C‐ and N‐termini are crucial for both trafficking from the endoplasmic reticulum, as well as post‐Golgi. Furthermore, for correct oligomerization of the channel, the proximal and distal parts of the C‐terminus of the channel are crucial (Roncarati et al. 2005).

CaM is encoded by three genes (*CALM1*, *CALM2*, and *CALM3*) that are translated into the exact same protein sequence. The sentence should be as follow: The three genes are all expressed in the human heart, where *CALM3* in one study was found to be the highest expressed followed by *CALM2* and *CALM1* (Crotti 2013). CaM serves numerous functions within the cells and is found to be almost 100% conserved among chordates and between the three genes (Berchtold and Villalobo 2014). Hence, it has until recently been assumed unlikely that mutations in the CaM encoding genes would occur (Marshall 2015). However, the recent research has led to the discovery of several CaM mutations in a small group of patients presented mainly with cardiac specific phenotypes, such as long QT syndrome (LQTS), idiopathic ventricular fibrillation (IVF), and catecholaminergic polymorphic ventricular tachycardia (CPVT). The vast majority of these mutations are de novo mutations, in line with the fact that they drastically increase the mortality (Nyegaard 2012; Crotti 2013; Makita 2014; Marsman 2014; Reed 2015; Pipilas 2016; Jimenez‐Jaimez 2016; Chaix 2016; Gomez‐Hurtado and Novel 2016). Since the role of SK channel current becomes more evident in the ventricles under conditions with increased sympathetic activation, we find it highly relevant to investigate the effect of these novel arrhythmogenic CaM variants on SK3 channel function by evaluating their impact on both channel gating and membrane trafficking.

## Methods

### Molecular biology

The rat CaM (*r*CaM) encoding DNA fragments from the pCS2 + expression vector (Berchtold 2016) were subcloned into the dual‐function plasmid vector pXOOM (Jespersen 2002). All CaM variants were PCR amplified and subcloned into the BamHI and HindIII sites of pXOOM. All PCRs were performed with Pfu Ultra II Fusion HS DNA Polymerase (Agilent Technologies, USA) under buffer conditions as proposed by the supplier with initial denaturation at 95°C for 2 min followed by 15 rounds of denaturation at 95°C for 30 sec, annealing at 50°C for 10 sec and elongation at 72°C for 1 min. The template plasmid, pCS2 +, was digested with DpnI (Thermo Fisher Scientific, USA), and the PCR products were purified using the NucleoSpin^®^ Gel and PCR Clean‐up Kit (MACHEREY‐NAGEL, Germany). The transformation was performed into *E.* *coli* DH5α^™^ (Thermo Fisher Scientific, USA) using the heat shock method. 16–18 h after transformation, plates were screened for positive colonies and purified using the NucleoBond^®^ Xtra Midi kit for plasmid DNA purification (MACHEREY‐NAGEL, Germany). pXOOM‐*r*CaM^WT^ was used as template in order to generate variant pXOOM‐*r*CaM^A103V^ by site directed mutagenesis using PCR and mutated oligonucleotide primers (Eurofins Genomics, Germany). The PCR was performed with the same conditions as mentioned above as well as the transformation and purification of the plasmid.

The integrity of all plasmids was verified by complete DNA sequencing of the cDNA insert (Macrogen Europe, Netherlands).

### Cell biology and transfection

Acesion Pharma (Copenhagen, Denmark) kindly provided Human Embryonic Kidney 293 (HEK293) cells stably expressing human SK3 (*h*SK3) used for the patch‐clamp experiments (HEK293‐*h*SK3). The cells were cultured in Dulbecco’s modified Eagles medium 1965 (DMEM1965) supplemented with NaHCO3, HEPES, and GlutaMAX. Additionally, the medium was supplemented with 10% FBS (Sigma‐Aldrich, Denmark), 1% pen/strep and 100 *μ*g/mL geneticin (Thermo Fisher Scientific, USA) and the cells were grown at 37°C in a humidified atmosphere with 5% CO_2_. HEK293‐*h*SK3 cells grown to 70% confluence in a T25 flask were co‐transfected with 1 *μ*g of pXOOM‐*r*CaM^WT or variant^ and 0.1 *μ*g of the enhanced Green Fluorescent Protein (eGFP) for easy identification of transfected cells using Lipofectamine^TM^ 2000 (Thermo Fisher Scientific, USA) according to the manufacturer’s protocols. Patch clamp experiments were performed 24 hr ± 4 after transfection.

For the immunocytochemistry experiments Madin‐Darby Canine Kidney (MDCK) cells were used and grown in DMEM1965 supplemented with 100 U/mL penicillin, 100 mg/mL streptomycin and 10% FBS (Sigma‐Aldrich, Denmark) at 37°C in a humidified atmosphere with 5% CO_2_. MDCK cells were co‐transfected in suspension with 1 *μ*g of pXOON‐*r*SK3 and 1 *μ*g of pXOOM‐*r*CaM^WT or variant^ using Lipofectamine ^TM^ 2000 (Thermo Fisher Scientific, USA) according to the manufacturer’s protocols. After transfection, the cells were plated on glass coverslips (12 mm in diameter, Thermo Scientific) and grown to confluence before fixing the cells.

### Electrophysiological recordings

The coverslips with cells were transferred to the recording chamber and perfused with high K^+^ solution consisting of (in mmol/L): 150 KCl, 0.1 CaCl_2_, 3 MgCl_2_, and 10 N‐(2‐ hydroxyethyl)piperazine‐N’‐(2‐ethanesulfonic acid) (HEPES), 10 glucose and 0.1% bovine serum albumin (BSA); pH 7.4 was adjusted with 10 mol/L KOH to keep the total concentration of K^+^ at 154 mmol/L corresponding to the K^+^ concentration in the patch pipette. Pipettes were filled with a solution consisting of (in mmol/L): 108 KCl, 15 KOH, 1.167 MgCl_2_, 31,25/10 KOH/EGTA, 10 HEPES and CaCl_2_ to yield a calculated free Ca^2+^ concentration of 400 nmol/L. The whole‐cell Ca^2+^ activated SK current, I*_SK_,_Ca_*, was acquired at room temperature upon application of voltage ramps from −100 mV to +60 mV from a holding potential of −80 mV. Every 5 sec, ramps of 200 msec in duration were applied. Upon current stabilization 1 *µ*mol/L of apamin (Alomone Labs, Jerusalem, Israel), a selective SK channel blocker, was used to completely inhibit the SK current.

### Immunocytochemistry

Transiently transfected MDCK cells grown to a confluent, polarized state on glass coverslips were fixed in 4% paraformaldehyde in PBS for 30 min at room temperature. Quenching was performed by 30 min incubation with 0.2% BSA in PBS supplemented with 0.1% Triton X‐100 (PBST). The cells were incubated for 1 hr in primary antibodies diluted in PBST and secondary antibodies, also diluted in PBST, were applied for 45 min. The coverslips were washed twice in PBS supplemented with 0.1% Triton X‐100 and once in PBS before they were mounted in Prolong Gold Antifade reagent (Thermo Fisher Scientific, USA). Primary antibodies used: rabbit anti‐K_Ca_2.3 (SK3) (N‐term) (1:200) (Alomone Labs, Jerusalem, Israel) and mouse anti‐FLAG^®^ M2 (1:500) (Sigma‐Alderich, Brondby, Denmark). Secondary antibodies used: Alexa Flour^®^ 568‐conjugated goat‐anti‐rabbit IgG (1:800) and Alexa Fluor^®^ 488‐conjugated donkey anti‐mouse IgG (1:200). DAPI (1:300) was used to stain the nucleus (Thermo Fisher, USA).

Laser scanning confocal microscopy was performed using the Zeiss LSM 710 confocal system. The operator was blinded to the imaging of the samples. Images were acquired using a 63× oil immersion objective, numerical aperture 1.4 with a pinhole size of 1AU and a pixel format of 1024 × 1024. Alexa Fluor 488 dye was excited by the 488 nm spectral line of argon‐ion laser, Alexa Fluor 568 was excited by 561 nm diode laser and DAPI by the 405 nm laser line. Images were processed using Zen Lite 2011 and Zen Edition 2011.

### Data analysis

**Figure 1:**
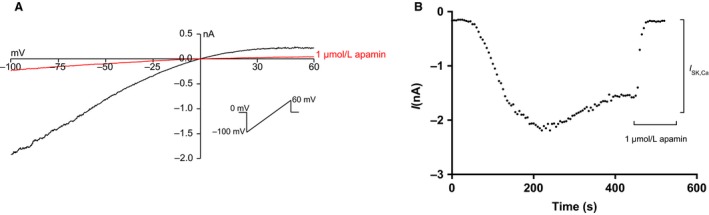
Ca^2+^ dependent activation of the whole‐cell *I* *_SK,Ca_*. (A) Representative traces showing the whole‐cell current from a HEK293‐*h*SK3 (SK3) before and after application of 1 *µ*mol/L apamin. Currents were recorded in symmetrical K^+^ with a free Ca^2+^ concentration in the pipette of 400 nmol/L. Voltage ramps from −100 mV to +60 mV from a holding potential of −80 mV were applied. Ramps of 200 msec were applied every 5 sec. (B) Whole‐cell currents recorded in the corresponding SK3 cell at −80 mV and plotted as a function of time. SK3 activity was allowed to stabilize in the SK3 cell before the bath solution was shifted to a 1 *μ*mol/L apamin‐containing bath solution, at the time indicated by the bar, which lead to a complete block of the SK3 current.

All the *I*_SK_ traces recorded were processed and analyzed in Igor Pro 4.04 (Wavemetrics, USA). The *I*_SK_ current was defined as the apamin‐sensitive current as indicated in Figure 1B. The current data are presented as pA/pF with the CaM^WT^ considered as the control sample and assigned the value of 1. For the quantification of the confocal images ImageJ was used. A line was drawn across the cell and the distance (pixels) was plotted as a function of the grey value. The mean grey value for the membrane area was divided with mean grey value for the intracellular compartments to obtain the SK3 ratio. All the analyse were performed blinded.

Data are presented as mean ± standard error of the mean (SEM). Statistical analysis was done using One‐way analysis of variance (ANOVA) with either Tukey or Dunnett’s post hoc analysis as indicated in the relevant figure legends. In all statistical analysis, the level of significance was 95% which means that *P*‐values <0.05 were considered to be statistically significant. GraphPad Prism ed. 7 (GraphPad Software, USA) was used for statistical analysis. The statistical significance in figures is denoted by *P < 0.05, **P < 0.01, ***P < 0.001, and ****P < 0.0001.

## Results

CaM mutations have been reported in all three *CALM* genes and the vast majority of these are de novo mutations. All CaM mutations identified so far are located in the C‐lobe of CaM except from CaM^N54I^ and CaM^F90L^, which are located in the N‐lobe and linker region, respectively (Marshall 2015).

Whole‐cell patch clamp recordings were conducted in HEK293 cells stably expressing *h*SK3 (HEK293‐*h*SK3). Figure 1 shows a representative current‐voltage relationship and the corresponding whole‐cell current at −80 mV plotted as the function of time. The SK channel mediated K^+^ currents are activated by a rise in intracellular Ca^2+^. In approximately 20% of the cells a run‐down was observed before the current was stabilized. This rundown‐behavior of SK current has been observed before (Strobaek 2000). The reason for this is still unclear, but a possible explanation could be desensibilisation of the interaction between CaM and the CaMBD of SK channels. The run‐down was observed both in cells expressing CaM^WT^ and in cells expressing mutant CaM, and an equal fraction of cells exhibited this run‐down behavior across groups. After current stabilization, addition of 1 *μ*mol/L apamin reduces the *I*_SK,Ca_ significantly, confirming that SK3 channels are potently blocked by apamin (Fig. 1).

**Figure 2:**
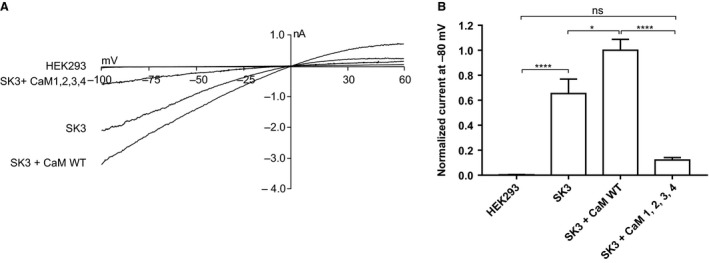
Effect of CaM^WT^ and CaM^1,2,3,4^ on *I* *_SK,Ca_*. (A) Representative traces showing the whole‐cell current from non‐transfected HEK293 cells, HEK293‐*h*SK3 (SK3) cells and SK3 cells transiently transfected with either CaM^WT^ or CaM^1,2,3,4^. Currents were recorded in symmetrical K^+^ with a free Ca^2+^ concentration of 400 nmol/L in the pipette. Voltage ramps from −100 to +60 mV from a holding potential of −80 mV were applied. Ramps of 200 msec were applied every 5 sec. (B) Normalized current densities as measured in non‐transfected HEK293 cells, HEK293‐hSK3 (SK3) cells and SK3 cells transiently transfected with either CaM^WT^ or CaM^1,2,3,4^ at −80 mV. As compared to the SK3 cells, transfection with CaM^WT^ significantly increases the currents, while transfection with CaM^1,2,3,4^ leads to reduction in the current. The current data are presented as pA/pF with the CaM^WT^ considered as the control sample and assigned the value of 1. Analysis was performed using one‐way ANOVA with Tukey’s post hoc analysis.

Overexpressing CaM^WT^ in the HEK293 cells resulted in a significant up‐regulation of *I*_SK,Ca_ (*P < 0.05)* (Fig. 2) indicating that CaM is a limiting factor for SK3 channel function. We further evaluated the importance of the Ca^2+^‐CaM interaction for the SK3 channel gating by expressing a CaM variant (CaM^1,2,3,4^) that has all 4 Ca^2+^‐binding sites mutated and hence is unable to bind Ca^2+^. This led to a profound down‐regulation of *I*_SK,Ca_ when compared with CaM^WT^ (*P* < 0.0001) (Fig. 2).

**Figure 3:**
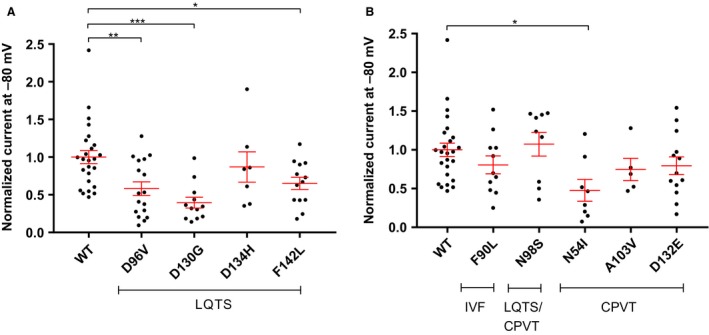
Impact of CaM variants on *I* *_SK,Ca_*. Obtained current densities for HEK293‐*h*SK3 cells transiently transfected with (A) LQTS CaM variants or (B) IVF, LQTS/CPVT, and CPVT variants. The current densities represent the apamin sensitive current measured at −80 mV. Currents were recorded in symmetrical K^+^ with a free Ca^2+^ concentration in the pipette of 400 nmol/L and upon application of voltage ramps from −100 to +60 mV from a holding potential of −80 mV. Ramps of 200 msec were applied every 5 sec. The current data are presented as pA/pF with the CaM^WT^ considered as the control sample and assigned the value of 1. Statistical analysis was performed using one‐way ANOVA with Dunnett’s post hoc analysis.

In order to evaluate the impact of the novel CaM variants on *I*_SK,Ca_, HEK293‐*h*SK3 cells were transiently transfected with the CaM variants and current measured. The LQTS‐associated variants CaM^D96V^, CaM^D130G^, and CaM^F142L^ reduced *I*_SK,Ca_ compared with CaM^WT^ *(P* < 0.01*, P* < 0.001, and *P* < 0.05, respectively*)* (Fig. 3A)_._ The LQTS‐associated variant CaM^D134H^ had a tendency toward decreasing the current, albeit not significantly (Fig. 3A). The CPVT associated variants CaM^N54I^ also reduced the *I*_SK,Ca_ compared with CaM^WT^ (*P* < 0.05) (Fig. 3B). The other CPVT‐associated variants CaM^A103V^ and CaM^D132E^ had a tendency toward decreasing the current, albeit not significantly (Fig. 3B). Variants CaM^F90L^ and CaM^N98S^ did not have any impact on *I*_SK,Ca_ conducted through SK3 channels (Fig. 3B).

**Figure 4:**
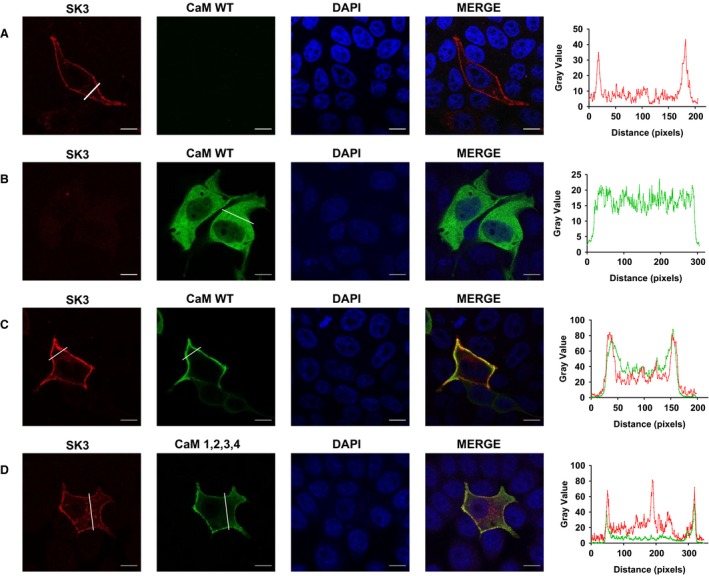
Expression and distribution of SK3 channels and CaM^WT^ in MDCK cells. Confocal images of MDCK cells transiently transfected with either (A) SK3 channels (B) CaM^WT^ (C) SK3 channels or co‐transfected with CaM^WT^ or (D) CaM^1,2,3,4^. The white line shows where in the cell the line scan was performed. Scale bar 10 *µ*m.

**Figure 5:**
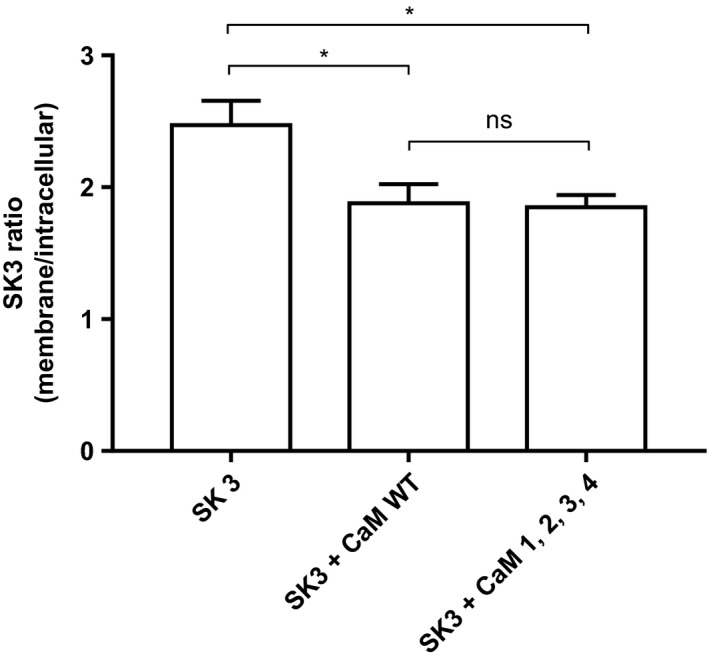
Quantified SK3 signals. Quantification of the confocal signals. Data are expressed as a ratio between the membrane and intracellular signals. Analysis was performed using one‐way ANOVA with Tukey’s post hoc analysis.

Next, we aimed at evaluating the effect of the CaM variants on trafficking and membrane location of the SK3 protein using MDCK cells. These cells have been widely used for studying trafficking of membrane proteins (Andersen 2011) and according to our immune‐analyses they do not endogenously express SK3 protein (data not shown). All the CaM proteins we studied were FLAG‐tagged, so we were able to specifically study exogenously introduced proteins. We expect that endogenous WT CaM also will form complexes with SK channels, but in competition with the highly expressed CaM variants. When overexpressing the SK3 alone, channel proteins were solely located at the cell membrane (Fig. 4A). On the other hand, when overexpressing CaM^WT^ alone, proteins were found to be distributed in intracellular compartments (Fig. 4B). This is not surprising taking into consideration all the interacting partners of CaM in the cell. When co‐expressing SK3 and CaM^WT^ SK3 remained primarily located in the surface membrane, were also CaM^WT^ now almost exclusively was found to be located with SK3 (Fig. 4C). The same distribution patterns were also observed when SK3 and CaM^1,2,3,4,^ were co‐expressed. This indicates that binding of Ca^2+^ to CaM is not necessary for correct trafficking and membrane localization of the SK3 protein (Fig. 4D). In addition, we found that the ratio of membrane to intracellular localized SK3 was reduced when co‐expressing CaM^WT^ and CaM^1,2,3,4^ (Fig. 5) (*P* < 0.05). However, this could be an artifact of the expression system caused by the inability of the folding and/or trafficking machineries in the cell to keep up with the over‐expression of the transfected contructs.

**Figure 6:**
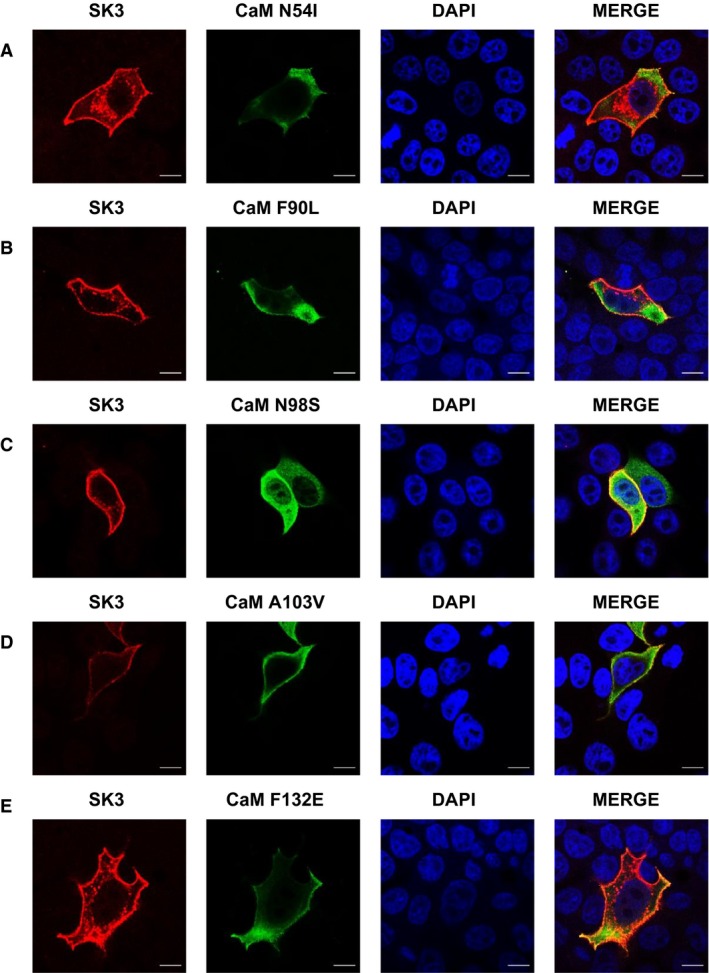
Expression and distribution of SK3 and LQTS‐associated CaM variants in MDCK cells. Confocal images of MDCK cells transiently co‐transfected with SK3 and CaM^variants^. Scale bar 10 *µ*m.

**Figure 7:**
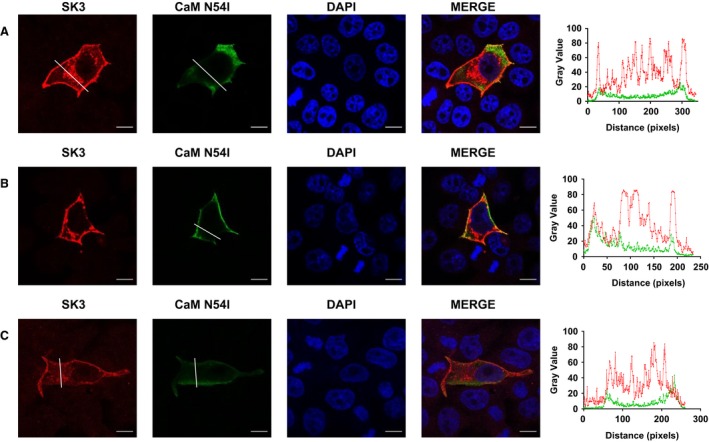
Expression and distribution of the SK3 and IVF/CPVT‐associated CaM variants in MDCK cells. Confocal images of MDCK cells transiently co‐transfected with SK3 and CaM^variants^. Scale bar 10 *µ*m.

**Figure 8:**
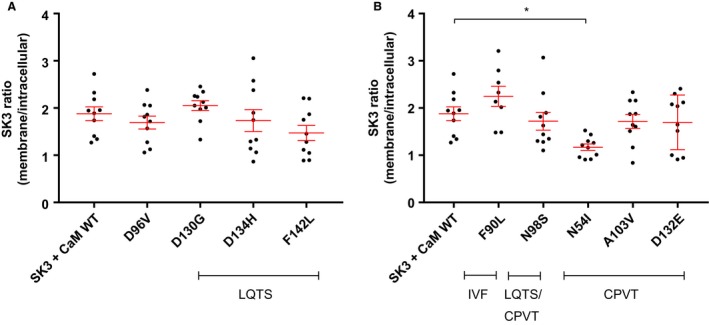
Expression and distribution of SK3 channels and CaM^N54I^ in MDCK cells. (A–C) Confocal images of MDCK cells transiently transfected with SK3 channels and CaM^N54I^ and the according line scans. The white line shows where in the cell the line scan was performed. Scale bar 10 *µ*m.

**Figure 9:**
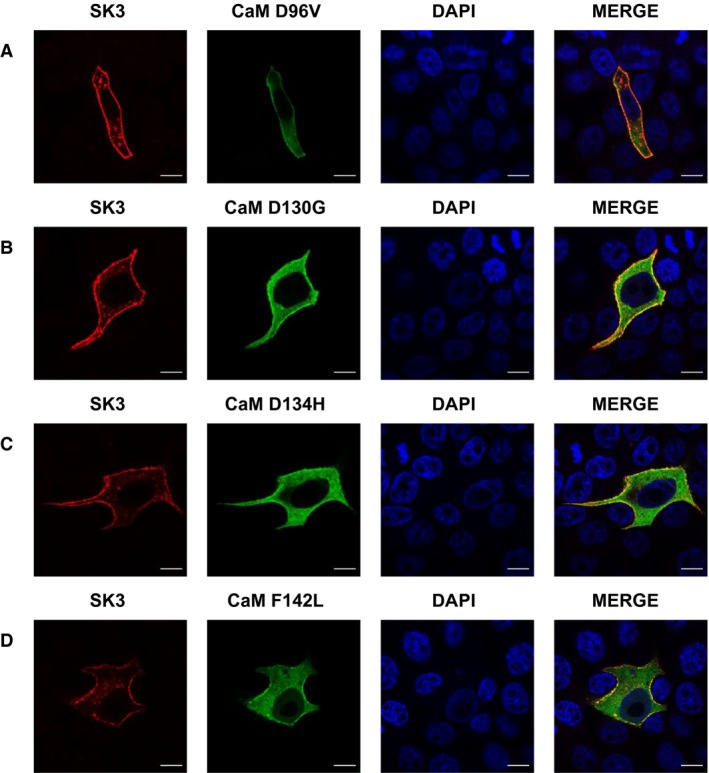
Quantified SK3 signals. Quantification of the confocal signals. Data are expressed as a ratio between the membrane and intracellular signals. (A) LQTS‐associated variants and (B) IVF/CPVT‐associated variants. Statistical analysis was performed using one‐way ANOVA with Dunnett’s post hoc analysis.

The localization studies reveal that the CaM variants have different impacts on the trafficking and surface localization of the SK3 protein. Co‐expressing CaM^N54I^ and SK3 results in both increased SK3 protein in intracellular compartments (Figs. 7, 8 and 9B) and decreased *I_SK,Ca_* (Fig. 3B). This result suggests that the decreased current observed when co‐expressing CaM^N54I^ (Fig. 3B) could be the consequence of incorrect membrane trafficking of SK3 rather than channel activation dysfunction, while the other variants that reduce the *I*_SK_ (CaM^D96V^, CaM^D130G^, CaM^F142L^) most likely render the SK3 channel non‐functional, as they still are present at the surface membrane (Figs. 6 and 9A).

## Discussion

Since the first human CaM mutations were published in 2012 by *Nyegaard et al.,* several of other CaM mutations have been described over the years in patients with severe cardiac arrhythmias (Nyegaard 2012). The molecular mechanisms leading to these cardiac arrhythmias are still largely unknown, but it has become increasingly evident that many different regulatory pathways and interacting proteins are involved (Jensen 2018). It is well known that CaM plays a crucial role as an intracellular Ca^2+^ sensor and is of high importance for a number of intracellular Ca^2+^ regulating proteins, including ion channels such as L‐type Ca^2+^ channels, Ca_V_1.2, RyR2, and SK channels. The altered Ca^2+^ handling could potentially result in a pathological condition in the ventricles where SK channels have a functional role. We therefore sought to investigate the functional impact of a range of CaM variants on SK3 channel by evaluating their effect on both channel gating and membrane trafficking.

### Role of SK channels in the pathogenesis of CaM‐associated arrythmias

As SK channels appears to play a role in some pathological conditions in the ventricles (Gui 2013; Bonilla 2014; Hundahl 2017; Chen 2018; Hamilton 2019), it could be speculated that the SK channels also have a contributory role in the pathogenesis of CaM‐associated LQTS. Three out of the four LQTS‐associated CaM variants significantly down‐regulated the SK current, with CaM^D130G^ being the variant most profoundly decreasing SK current with no effect on the trafficking of the channel. Variant D130G decreases the affinity of the C‐domain of CaM for Ca^2+^, but have little or no effect on Ca^2+^ binding to the N‐domain (Søndergaard et al. 2015). In the SK/CaM complex Ca^2+^ only binds to the N‐domain of CaM and as this interaction is not affected, the data suggest that this variant most likely loses its ability to afflict conformational changes, which potentially affects the function of the channel. *Berchtold* et al. showed that out of six CaM variants tested, CaM^D130G^ had the most prominent effect on Ca^2+^/CaM‐dependent kinase II and also induced bradycardia in zebra fish (Berchtold 2016). In general, the LQTS phenotype is manifested by a QT interval prolongation resulting from a prolonged cardiac action potential caused by either increased inward depolarizing currents (*I*_Na _and *I*_Ca_) or decreased outward repolarizing currents (*I*_Kr_, *I*_Ks_, and *I*_K1_) (Giudicessi and Ackerman 2012). A reduction in the SK current could be speculated to decrease the outward K^+^ current and contribute to a prolongation of the QT interval. *Yu* and colleagues investigated the putative role of SK channels in LQTS, showing that SK channel blockage of murine hearts with a pharmacologically induced reduced repolarization capacity leads to prolongation of the QT interval. This indicates that suppression of the SK current can contribute to the long QT phenotype (Yu 2016). However, when using mouse models, electrophysiological parameters should be done with caution as the cardiac ion channel composition and the heart rate differs from larger species. However, what is known from several species is that LQTS‐associated CaM variants have been shown in several studies to increase Ca^2+^ influx by suppressing Ca^2+^‐CaM‐dependent inactivation of the L‐type Ca^2+^ channels, which would lead to increased SK current (Limpitikul 2014; Yin 2014; Pipilas 2016). Increased intracellular Ca^2+^ may in addition activate NCX, which would result in further inward current.

In the study by *Yu et al.* five of the same CaM variants were studied (CaM^N54I, N98S, F90L, D96V and D130G^). Unlike what we found in our study on SK3 channels, all of the variants studied by *Yu et al.* gave rise to a significant reduction in SK2 current. This discrepancy could be explained by the unregulated ratio between endogenous CaM^WT^ and the CaM when using transient expression systems. Furthermore, *Yu et al.* did not find any impact on trafficking nor membrane expression by any of the CaM variants. HEK293 cells, such as those used for the immunocytochemistry studies by *Yu et al.*, are known to have a higher degree of intracellular ion channel accumulation in contrast to MDCK cells used in the present study. The high ratio of SK3 localized to the membrane in polarized MDCK cells facilitates low signal to noise ratio which is needed to clearly distinguish and quantitate the plasma membrane/cytosol population of SK3. Despite the discrepancies, our study does support the findings by *Yu et al.* confirming that variant CaM^N54I^, CaM^D96V^, and CaM^D130G^ leads to reduced SK channel current (Yu 2016).

CaM^N54I^, which is one out of the three CPVT‐associated variants studied, down‐regulated the SK current, and also gave rise to larger amount of SK3 located intracellularly with no co‐localization between SK3 and CaM^N54I^ protein indicating a lack of interaction between these two proteins. The N54I variant is unique, as it is the only mutant located in the N‐terminal lobe of CaM and it is not part of the Ca^2+^ coordination or the hydrophobic target binding patches. In line with this, with this, it has previously been found that the Ca^2+^‐binding affinity of CaM in neither the C‐ nor N‐domain is affected (Vassilakopoulou 2015). Most studies point toward a defective RyR2 interaction and regulation by CPVT‐associated CaM variants as being the underlying mechanism (Hwang 2014). Variant N54I demonstrates enhanced RyR2 affinity and as a result increases Ca^2+^ sparks and frequency of Ca^2+^ waves (Hwang 2014; Vassilakopoulou 2015). To conclude if the impact on the SK3 channels of this variant is involved in the development of CVPT will require further studies.

### Role of CaM and Ca^2+^ in SK channel trafficking and gating

A transient overexpression of CaM^WT^ in HEK293 cells that stably express the SK3 protein led to a significant increase in *I*_SK,Ca_ suggesting that CaM is a limiting factor in SK3 channel regulation. This is in line with what has been previously reported for SK2 channels (Yu 2016) and further supported by reports staiting that the number of CaM targets in the cell and their requirement for CaM is significantly higher than the total CaM concentration, which makes CaM a limiting factor in their regulation (Persechini and Stemmer 2002).

We also studied the quadruple CaM variant CaM^1,2,3,4^ which is unable to bind Ca^2+^. This mutant has been widely used to study the Ca^2+^‐dependent and –independent interactions of CaM with many different targets, among those also the SK channels (Lee 2003). In this study, we confirmed that the Ca^2+^ ‐independent interaction is sufficient for normal SK3 channel trafficking and membrane expression as normal surface expression and localization was observed, whereas the Ca^2+^‐dependent interaction is needed for gating and activation of the SK3 channel. Although we did find that this variant exerted a dominant negative effect, it did not cause complete loss of function, as would be expected. An SK channel complex contains four CaM molecules and it is possible that in a single complex there is a mixture of endogenous and variant CaM.

This study provides new insight into the dysfunction of mutant CaM in relation to SK3 channels. Understanding the relationship between both WT and mutant CaM and SK channel function is important when designing novel pharmacological compounds targeting the SK channels on order to obviate or treat arrhythmias. The unique Ca^2+^‐dependent and –independent interaction between CaM and the CaMBD of SK channels means that there are several possible molecular ways by which the CaM mutations can exert their effect on SK3 channels. Most likely, these arrhythmogenic variants disrupt different cellular pathways by distinct mechanisms through failed interactions/activation of several targets. The interaction between CaM and a given target protein will be determined by the position of the mutation within the CaM molecule and how it effects the conformational adaption and stability of the CaM molecule. Therefore, there is a need for studying the underlying molecular mechanisms leading to the cardiac arrhythmias and how the SK channels are involved in these.

## Conclusion

In conclusion, this study puts forward that several of the arrhythmogenic CaM variants, including CaM^N54I^, CaM^D96V^, CaM^D130G^, and CaM^F142L^ significantly downregulate the SK3 channel current. The underlying mechanisms seem to differ, as CaM^N54I^ affects the membrane expression while the other variants most likely affect channel gating, thereby limiting the functionality of the SK3 channel.

## Conflict of Interest

The authors have no conflict of interest to declare.
